# ^1^H NMR spectra dataset and solid-state NMR data of cowpea (*Vigna unguiculata*)

**DOI:** 10.1016/j.dib.2017.01.013

**Published:** 2017-02-03

**Authors:** Elenilson G. Alves Filho, Lorena M.A. Silva, Elizita M. Teofilo, Flemming H. Larsen, Edy S. de Brito

**Affiliations:** a EMBRAPA Agroindústria Tropical, Fortaleza, CE, Brazil; b LABIOTEC, Dept. Food Technology, Federal University of Ceará, Brazil; c Center of Agricultural Science, Federal University of Ceará, Fortaleza, CE, Brazil; d Department of Food Science, University of Copenhagen, Denmark

**Keywords:** Cowpea seeds, ^1^H *q*NMR, Chemometrics, CP-MAS, SP/MAS

## Abstract

In this article the NMR data from chemical shifts, coupling constants, and structures of all the characterized compounds were provided, beyond a complementary PCA evaluation for the corresponding manuscript (E.G. Alves Filho, L.M.A. Silva, E.M. Teofilo, F.H. Larsen, E.S. de Brito, 2017) [3]. In addition, a complementary assessment from solid-state NMR data was provided. For further chemometric analysis, numerical matrices from the raw ^1^H NMR data were made available in Microsoft Excel workbook format (.xls).

**Specifications Table**

**Table:** 

Subject area	*Analytical chemistry*
More specific subject area	^*1*^*H NMR combined with chemometrics and solid-state NMR*
Type of data	*Tables and figures*
How data was acquired	*NMR spectrometer Agilent 600-MHz, 5 mm (H-F/*^*15*^*N-*^*31*^*P) One Probe™*
Data format	*Raw and analyzed*
Experimental factors	*Seeds were peeled and pulverized.*
	*Liquid-state NMR analysis: 15 mg was soaked in 400 μL of D*_*2*_*O, 200 μL of phosphate buffer pH 4.3 and 1.0 mM of TMSP-d*_*4;*_*automatic mixed (5 min) at room temperature, centrifuged at 6000 rpm.*
	*Solid-state NMR analysis: 50–55 mg were inserted in the Kel-F NMR rotor of 5 mm.*
Experimental features	^*1*^*H NMR acquisition: PRESAT pulse sequence; 90° calibrated pulse; 128 scans, 64k of time domain points; spectral window of 15 ppm, acquisition time of 6.7 s; relaxation delay of 15.0 s; temperature of 298 K.*
	^*1*^*H NMR data processing: Lorentzian broadening of 0.3 Hz, zero filling to 64k points.*
Data source location	*Fortaleza-Ceará, Brazil, cowpea germplasm bank at Federal University of Ceará*
Data accessibility	*Data was provided in the article and raw data was provided as.xls*
Related research article	*Genotype evaluation of cowpea seeds (Vigna unguiculata) using*^*1*^*H qNMR combined with exploratory tools and solid-state NMR*

**Value of the data**

• The NMR data (chemical shifts and coupling constants) and structures may be helpful to other NMR spectroscopists in the assignment of signals in complex matrices as food.
• Useful to be used as reference for the characterization of organic compounds through NMR.
• Numerical matrices from the raw ^1^H NMR data were made available for complementary evaluation, or construction of NMR database, or useful for the development of new chemometric algorithms.
• The data provide a comprehensive and complementary comparison among different genotypes of cowpea seeds using ^1^H-NMR combined with chemometrics and solid-state NMR.

## 1 Data

**Fig. 1:**
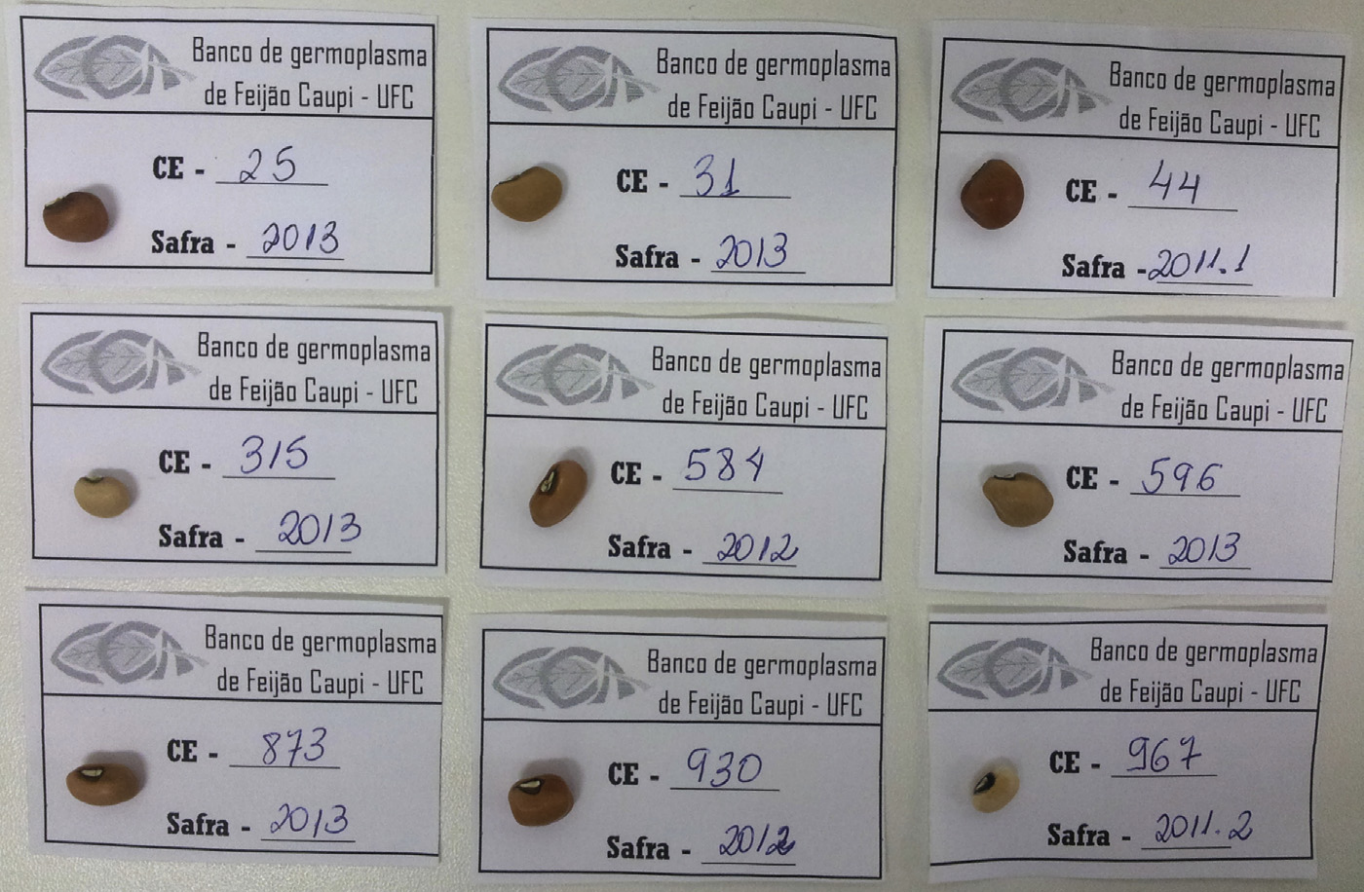
PC1 *vs.* PC3 scores (left side) and loadings (right side) coordinate system for different cultivars of cowpea analysing only aromatic region.

**Fig. 2:**
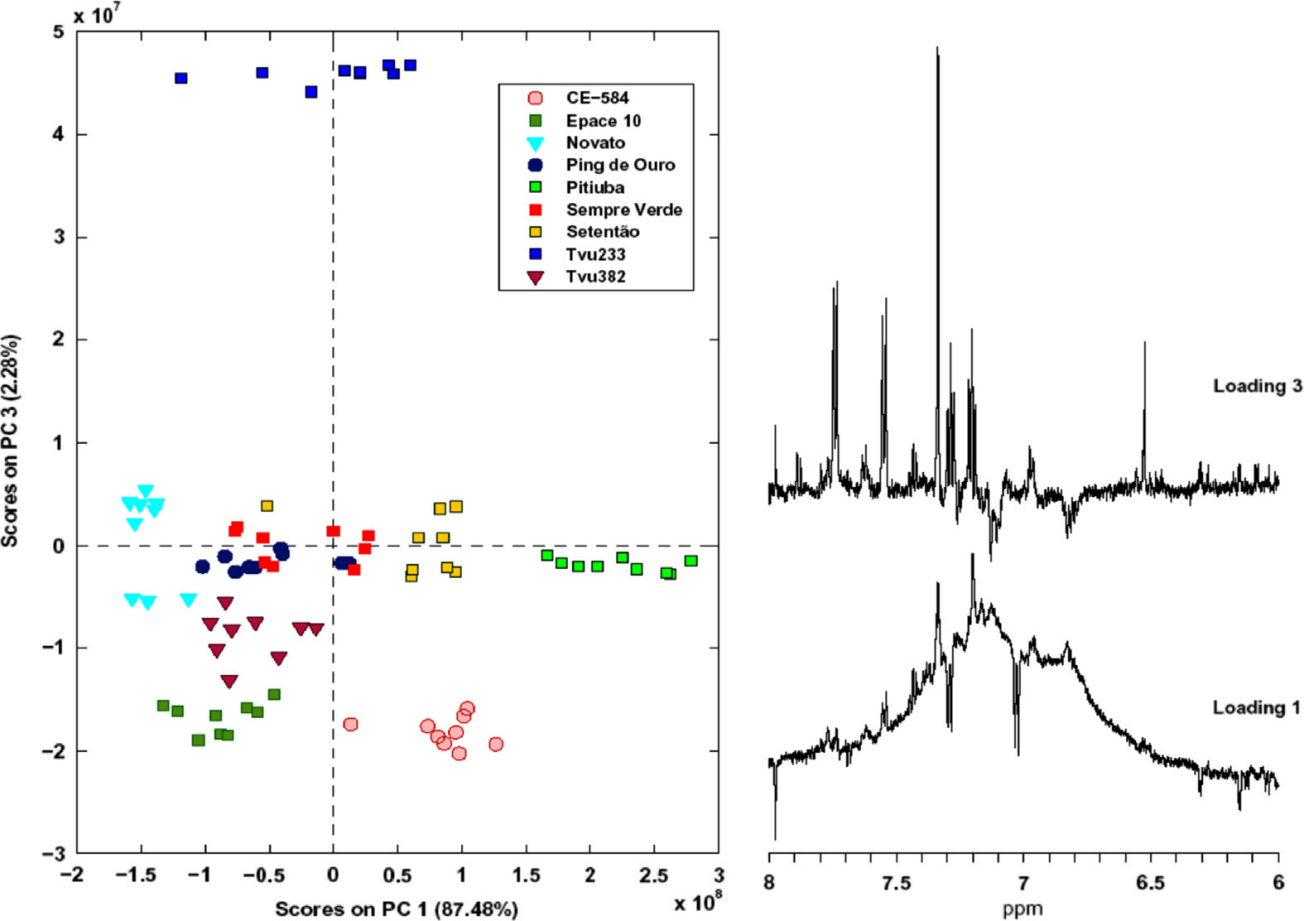
^13^C CP-MAS spectra of the cowpea seed with a) Sempre Verde; b) Tvu 233; c) Pitiuba; d) Novato; e) CE-584; f) Setentão; g) Pingo de Ouro; h) Tvu 382; i) Epace 10.

**Fig. 3:**
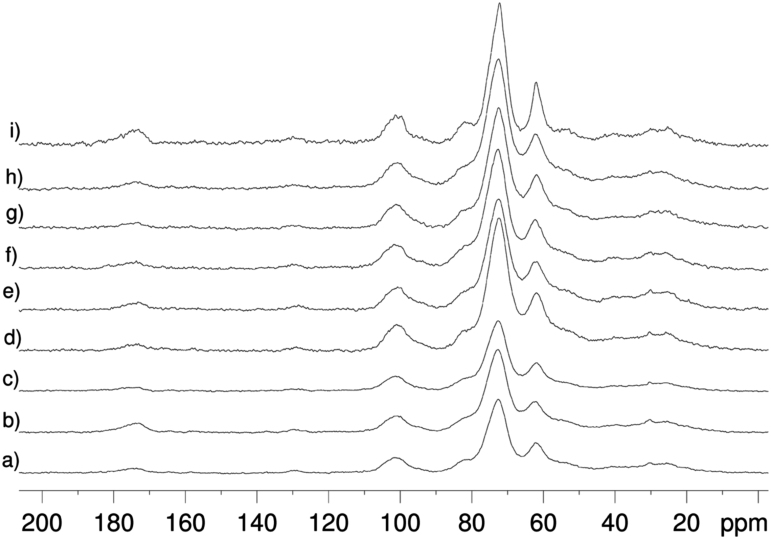
^13^C SP-MAS spectra of the cowpea seed with a) Sempre Verde; b) Tvu 233; c) Pitiuba; d) Novato; e) CE-584; f) Setentão; g) Pingo de Ouro; h) Tvu 382; i) Epace 10.

Table 1 presents the morphoagronomic characteristics of the cowpea seeds. Table 2 illustrates the structures of the 30 compounds identified in cowpea seeds with the corresponding ^1^H and ^13^C NMR chemical shifts, multiplicity, and constant coupling [4], [5], [6], [7], [8], [10]. PC1 vs. PC3 scores and loadings coordinate system for different cultivars of cowpea evaluating only the aromatic region are presented in Fig. 1. Fig. 2, Fig. 3 show the comparison of the ^13^C CP-MAS and the ^13^C SP-MAS spectra of the cowpea seeds [3].

## 2 Experimental design, materials and methods

**Fig. 4:**
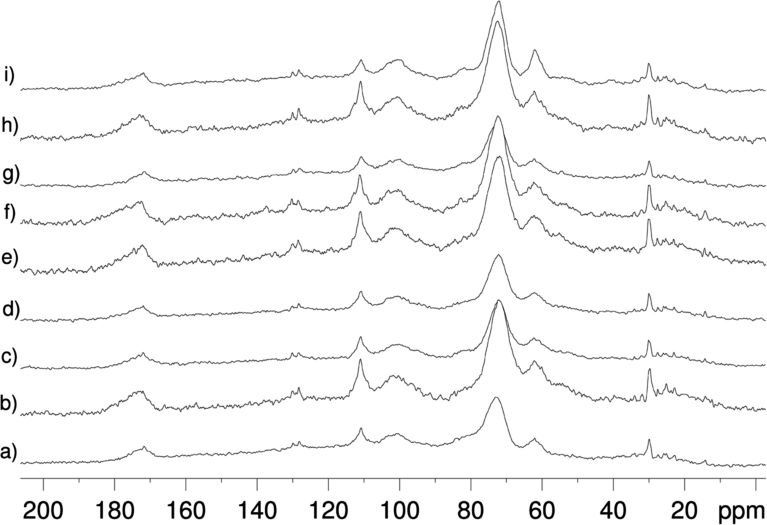
Nine seeds of cowpea (*Vigna unguiculata*).

Fig. 4 presents nine cowpea seeds from the germplasm bank of the Center of Agricultural Science at Federal University of Ceará (CCA/UFC), Brazil, with the accession numbers and the vintage years.

### 2.1 ^1^H NMR analysis

The NMR experiments were performed on an Agilent 600-MHz spectrometer equipped with a 5 mm (H-F/^15^N-^31^P) inverse detection One Probe™. The ^1^H NMR spectra were acquired under quantitative parameters using the PRESAT pulse sequence for water suppression, since this pulse sequence presented the best irradiation profile for quantitative determination of the signals near of the water suppression region [9]. The data were acquired with the RF pulse calibrated to 90° and 128 scans, 64 k of time domain points for a spectral window of 15 ppm, acquisition time of 6.7 s and a relaxation delay of 15.0 s. The temperature was 298 K. The spectra were processed by applying exponential Lorentzian broadening of 0.3 Hz and zero filling to 64k points before Fourier transformation. Phase correction was performed manually for each spectrum and the baseline correction was applied over the entire spectral range. All spectra were referenced to the TMSP-d_4_ resonance at 0.0 ppm.

### 2.2 Matrices from the ^1^H NMR data

Two matrices were used for chemometric evaluation: Table 3 for PCA (Principal Component Analysis); Table 4 for clustering analysis. For the construction of the Table 3, all the ^1^H NMR data were converted to American Standard Code for Information Interchange (ASCII) files and imported to Microsoft Excel software (Elenilson G. [2]). For the construction of the Table 4, each spectrum was divided into 0.04 ppm wide buckets, using simple rectangular bucket, sum of intensities in integration mode and scaled to total intensity in scaling process (Elenilson G. [1])*.*

## Figures and Tables

**Table 1:** Morphoagronomic characteristics of the nine seeds of cowpea.

**Register number**	**Access name**	Color	Texture	Shape	Weight
**CE-25**	Sempre Verde	Green	Flat	Rhomboid	12.7
**CE-31**	Pitiuba	Brown	Flat	Reniform	19.4
**CE-44**	Novato	Brown	Flat	Rhomboid	21.8
**CE-315**	Tvu 233	Green	Flat	Ovoid	12.9
**CE-584**	CE-584	Brown	Flat	Reniform	23.0
**CE-596**	Setentão	Green	Flat	Rhomboid	16.9
**CE-873**	Epace 10	Brown	Flat	Rhomboid	19.4
**CE-930**	Pingo de Ouro	Brown	Flat	Rhomboid	19.8
**CE-967**	Tvu 382	Black - White	Flat	Ovoid	8.7

**Table 2:** Organic compounds identified in cowpea seeds.

Compounds /Structures	δ ^1^H	δ ^13^C	Ref.	Ref.
	(multip.*, J in Hz)		^1^H	^13^C
Amino Acids				
	3 – 1.42 (*d* 7.2)2 – 4.31 (o)	19.156.1	1.52 (*d,* 7.3)3.90 (*q*, 7.3)	19.153.4
			
			
	2,5 – 4.39 (o)3,4 – 2.86; 3.02 (o)	57.238.9	4.10 (*dd* 8.21, 3.91)3.18;3.38 (*ddd* 14.94, 8.21, 3.91)	56.140.5
			
			
	1 –	174.5		177.0
	5 – 2.17 (*s*)	17.7	2.10 (*s*)	16.6
	3 – 2.07	30.2	2.17 (*m*)	32.7
	4 – 2.39	34.1	2.63 (*t* 7.59)	31.6
	2 – 3.80	57.3	3.85 (*dd* 7.10; 5.38)	56.8
	2 – 3.51 (o)	o	3.57 (*d* 4.87)	63.5
	3 – 4.26 (o)	69.8	4.24 (*m*)	68.9
	4 – 1.33 (o)	22.3	1.32 (*d* 6.58)	22.3
	6 – 3.23	43.6	3.32 (*m*)	49.0
	5 – 1.71	29.3	1.99 (*m*)	26.4
	2 – 3.81	63.3	4.12 (*dd* 8.83; 8.42)	64.0
	3 – 2.20	29.4	2.34 (*m*)	31.7
	4 – 1.92	30.6	2.07 (*m*)	31.7
	5 – 3.24 (o)	43.6	3.23 (*t* 6.93)	43.3
	4 – 1.66 (*m*)	27.3	1.68 (*m*)	26.4
	3 – 2.17 (*m*)	29.4	1.91 (*m*)	30.5
	2 – 3.79 (o)	57.3	3.76 (*t* 6.11)	57.3
				
	2 – 3.62 (o)	o	3.82 (*d* 4.4)	n
	3 – 2.16 (o)	20.2	2.33 (*m*)	32.0
	4 – 0.91 (o)	21.7	1.02 (*d* 7.1)	19.1
	5 – 0.91 (o)	21.7	1.06 (*d* 7.1)	20.9
	2 – 3.81 (o)	46.8	3.55 (*s*)	44.3
	3 – 3.80	57.4	3.83 (*dd* 5.58; 3.80)	59.2
	2 – 3.83	63.2	3.95 (*m*)	63.1
	1 –	176.9		no
	2 – 4.01 (o)	54.3	3.90 (no)	55.1
	3 – 2.86; 3.00 (m)	38.8	2.71; 2.80 (no)	39.4
	4 –	175.8		no
	1 –	174.1		177.2
	2 – 3.80 (o)	57.3	3.74 (*dd* 7.19; 4.72)	57.6
	3 – 2.17 (o)	29.3	2.08 (*m*)	29.8
	4 – 2.54 (o)	34.8	2.34 (*m*)	36.3
	6,8 – 6.83 (*m*)	118.2	6.89 (*m*)	118.9
	5,9 – 7.10 (*m*)	133.1	7.19 (*m*)	133.5
	5,9 – 7.24 (*m*)	132.0	7.32 (*d* 6.98)	132.1
	6,8 – 7.42 (*m*)	131.8	7.42 (*m*)	131.8
	7 – 7.32 (*m*)	131.7	7.37 (*m*)	130.4
	8 – 7.84 (*m*)	119.0	7.71	121.2
	7 – 7.42 (*m*)	119.8	7.52	114.7
	5 – 7.33 (*m*)	125.6	7.30	127.9
	10 – 7.24 (*m*)	112.6	7.26	124.9
	9 – 7.10 (*m*)	122.2	7.19	122.2
	2 – o	o	4.04	57.9
	3 – o	o	3.46	29.1
	1 –	no	–	176.1
				
**Organic Acids**				
				
	3 – 1.32 (*d* 7.20)	21.7	1.37 (*d* 7.20)	22.9
	2 – 4.07 (o)	72.3	4.42 (*q* 7.20)	71.4
				
	4 – 2.88 (*m*)	39.2	2.99 (*t* 7,6)	42.2
	3 – 2.06 (*m*)	30.8	1.88 (*qui* 7,6)	26.3
	2 – 2.43 (*m*)	34.5	2.28 (*t* 7,6)	37.1
	1 –	no		166.2
	2 –	140.5		127.2
	3 – 9.10	148.4	8.97	152.8
	4 – 8.83	147.2	8.61	151.4
	5 – 8.07	130.3	7.54	123.3
	6 – 8.80	148.5	8.26	145.6
	1 –	181.2		184.1
	2 – 1.94 (*s*)	26.2	2.08 (*s*)	26.0
	1 – 8.48 (*s*)	no	8.39 (*s*)	172.4
	4,6 –	181.2		181.9
	3 – 2.58 (*d* 15.6)	47.6	2.68 (*d* 15.2)	45.5
	3 – 2.71 (*d* 15.6)	47.6	2.85 (*d* 15.2)	45.5
	2 – 4.44 (*m*)	69.2	4.28 (*m*)	73.2
				
	1 –			
	2 – 4.41	73.4	4.29	73.2
	3 – 2.85; 3.01	38.7	2.34; 2.65	45.5
	4 –			
				
	8,14 – 2.06	29.9	2.05	27.2
	2 – 2.38	34.0	2.34	34.0
	11 – 2.77	28.4	2.77	25.6
	10,12 – 5.30	130.8	5.33	128.1
	9,13 – 5.33	132.5	5.37	130.2
				
**Carbohydrates**				
				
	1 – 5.23 (o)	95.1	5.25 (*d* 3.80)	95.4
	2 – 3.47 (*m*)	72.3	3.89-3.36 (o)	72.2
	3 – 3.77 (*m*)	75.6	n	76.0
	4 – 3.56 (*m*)	74.0	n	72.8
	5 – 3.72 (*m*)	63.9	n	64.2
	6 – 3.85 (*m*)	75.5	n	74.5
				
	1 – 4.64 (o)	99.3	4.66 (*d* 8.10)	99.2
	2 – 3.26 (*m*)	77.5	3.25 (*t* 8.40)	77.6
	3 – 3.75 (*m*)	63.6	n	56.1
	4 – 3.48 (*m*)	78.8	n	79.0
	5 – 3.41 (*m*)	72.2	n	72.8
	6 – 3.90 (*m*)	63.7	n	63.1
				
	1 – 5.42 (*d* 3.7)	95.0	5.44 (*d* 3.8)	94.7
	3’ – 4.05 (*m*)	77,0	4.08 (*t* 8.4)	76,6
	4’ – 4.22 (*m*)	79,3	4.24 (*d* 9.0)	79,0
				
	1 – 5.02 (*m*)	101.1	4.98 (*d* 3.80)	101.1
	7 – 5.42 (*d* 3.81)	95.0	5.42 (*d* 3.85)	94.6
	15 – 4.24 (*m*)	79.3	4.22 (d 8.80)	79.9
				
	1 – 5.02 (*m*)	101.1	4.98 (*m*)	100.9
	7,13 – 5.44 (*d* 3.81)	95.0	5.42 (*d* 3.80)	94.8
	21 – 4.24 (*m*)	79.3	4.22 (d 8.80)	79.9
				
	1 – 5.02 (*m*)	101.1	4.98 (*m*)	100.9
	7,13,19 – 5.46 (*d* 3.81)	95.0	5.42 (*d* 3.80)	94.8
	3´´´´ – 4.24 (*m*)	79.3	4.22 (d 8.80)	79.9
				
Other Compounds				
	1 – 4.00 (o)	54.2	4.05 (*m*)	58.5
	3 – 3.19 (*s*)	56.5	3.19 (*s*)	56.7
	2 – 3.51 (o)	70.4	3.50 (*dd* 5.82; 4.16)	70.1
				
	2 – 5.91	105.2	5.79	103.7
	3 – 7.85	144.7	7.56	146.2

*s* – simplet; *d* – duplet; *t* – triplet; *q* – quadruplet; *quin* – quintet; *dd* – double duplet; o – overlapping signal; n – no information; no – not observed.
