# Supplementary material for: 1H NMR spectra dataset and solid-state NMR data of cowpea (Vigna unguiculata)
Source: Data Brief. 2017 Feb 3;11:136–46. doi: 10.1016/j.dib.2017.01.013 (PMC5310203; doi:10.1016/j.dib.2017.01.013)
Supplement: Supplementary file 2 — Supplementary material [file mmc1.doc]

Dear Journal Office,

I am sending you our manuscript entitled “**1H NMR spectra dataset and solid-state NMR data of cowpea (*Vigna unguiculata*)**” by Alves Filho, E. G. *et al*. We would like to have the manuscript considered for publication in the **Data in Brief** since to our knowledge, this is the first report in which non-targeted chemometric analysis of NMR data and solid state NMR were applied to investigate the variability of organic compounds in cowpea seeds. The NMR data may be helpful to other NMR spectroscopists in the assignment of signals in complex matrices as food, and the numerical matrices provided are useful for complementary evaluation, or construction of NMR database, or for the development of new chemometric applications.

Besides, we confirm that this manuscript has not been published elsewhere, is not under consideration by another journal and all authors have approved the manuscript and agree with its submission to **Data in Brief**.

I appreciate your time and consideration.

Yours Sincerely,

Elenilson de Godoy Alves Filho

**Authors**:

Elenilson G. Alves Filho^1,2^ – elenilson.godoy@yahoo.com.br

Lorena Mara A. Silva^1^ – lorena.mara@embrapa.br

Elizita M. Teofilo^3^ – elizita@ufc.br

Flemming H. Larsen^4^ – fhl@food.ku.dk

Edy S. de Brito^1^ – edy.brito@embrapa.br

**Affiliations and address:**

^1^EMBRAPA Agroindústria Tropical, Fortaleza-CE, Brazil.

^2^LABIOTEC, Dept. Food Technology, Federal University of Ceará-Brazil.

^3^Center of Agricultural Science, Federal University of Ceará, Fortaleza-CE, Brazil.

^4^Department of Food Science, University of Copenhagen, Denmark.
